# A Critical Assessment of Directed Connectivity Estimates with Artificially Imposed Causality in the Supramammillary-Septo-Hippocampal Circuit

**DOI:** 10.3389/fnsys.2017.00072

**Published:** 2017-09-29

**Authors:** Calvin K. Young, Ming Ruan, Neil McNaughton

**Affiliations:** ^1^Department of Psychology and Brain Health Research Centre, University of Otago, Dunedin, New Zealand; ^2^Wuhan Asia Heart Hospital, Wuhan, China

**Keywords:** directed connectivity, theta oscillations, hippocampus, septum, supramammillary nucleus, Granger causality, phase transfer entropy, phase slope index

## Abstract

Algorithms for estimating directed connectivity have become indispensable to further understand the neurodynamics between functionally coupled brain areas. The evaluation of directed connectivity on the propagation of brain activity has largely been based on simulated data or toy models, where various hidden properties of neurophysiological data may not be fully recapitulated. In this study, directionality was unequivocally manipulated in the freely moving rat in a unique dataset, where normal oscillatory interactions between the supramammillary nucleus (SuM) and hippocampus (HPC) were attenuated by temporary medial septal (MS) inactivation, and replaced by electrical stimulation of the fornix to evaluate the performance of several directed connectivity assessment methods. The directed transfer function, partial directed coherence, directed coherence, pair-wise Geweke-Granger causality, phase slope index, and phase transfer entropy, all found SuM to HPC theta propagation when the MS is inactivated, and HPC activity was driven by peaks of simultaneously recorded SuM theta. As expected from theoretical expectations and simulated data, signal features including coupling strength, signal-to-noise ratio, and stationarity all weakly affected directed connectivity measures. We conclude that all the examined directed connectivity estimates correctly identify artificially imposed uni-directionality of brain oscillations in freely moving animals. Non-auto-regressive modeling based methods appear to be the most robust, and are least affected by inherent features in data such as signal-to-noise ratio and stationarity.

## Introduction

To understand the brain it is crucial to understand how information is coded, represented and stored. This feat requires an understanding of how different parts of the brain are actively engaged and disengaged in functional circuits that allow behavioral output. There are many techniques, such as electro- and magneto-encephalograms (EEG/MEG), and functional magnetic resonance imaging (fMRI) used mostly in non-invasive human studies that allow us to gauge the timing of responses between spatially segregated areas in health and disease. By examining *if* (non-directed connectivity) and *how* (directed connectivity), brain activities are coupled, we gain further insight to how brain functions are organized and executed.

Correlational estimates between signals recorded from the brain have been a mainstay to infer information transfer, or at least functional engagement in the temporal domain, between brain circuits at different levels. Methods such as correlation, ordinary coherence, and phase synchronization have been used to infer a non-directed functional relation between signal sources. However, the direction of information transfer/activity modulation is crucial if we are to decode how information is transformed, used and stored at multiple levels of processing in the brain. The direction of connectivity can be inferred in various ways. In general, the most widely applied method for biological signals is based on the idea that if one signal's past can be used to minimize prediction errors on another's present/future, then the signal is said to “Granger cause” the other (Granger, 1969). Techniques such as partial directed coherence (PDC; Baccala and Sameshima, 2001), the directed transfer function (DTF; Kaminski and Blinowska, 1991), directed coherence (DCOH; Saito and Harashima, 1981), and Geweke-Granger causality (GGC; Geweke, 1982) are all implemented through auto-regressive modeling, where a transformation of model coefficients to the frequency domain, allowing the examination of Granger causality at different frequencies. Other methods such as phase transfer entropy (PTE; Lobier et al., 2014) share conceptual similarities to Granger causality (Barnett et al., 2009; Seghouane and Amari, 2012), as the implementation depends on the probability densities related to how much uncertainty can be accounted for by a signal's past alone, compared to the combination of all input signals' past. Phase slope index (PSI; Nolte et al., 2008) is a more mechanistic approach, based on the fact that if two signals are phase-locked, fluctuations in frequency should be linearly related to the phase lag/difference. Finally, *a priori* models using available anatomical and biophysical data to reduce the variance observed in the data such as structural equation (Astolfi et al., 2004) and dynamic causal modeling (David et al., 2006; Kiebel et al., 2008) are powerful approaches if the biological basis of the circuit studied is well-understood.

There have been many studies in the literature performing comparisons and benchmarks for various approaches in examining functional and directed connectivity (Astolfi et al., 2009; Florin et al., 2011; Silfverhuth et al., 2012; Fasoula et al., 2013; Wang et al., 2014), but most existing literature on the subject depend on simulated data that approximate biological data. Factors that may impact on the estimation of directed connectivity, such as the signal-to-noise ratio, are usually manipulated arbitrarily and without changes in other potential biases such as stationarity or non-linearity. In addition, benchmarking various algorithms on real data is usually focused on implementation alone rather than serving as a true test of performance on real biological systems; that is, the direction of interacting biological signal sources are rarely unequivocally known. Here, we use a unique dataset where theta oscillations in the rodent hippocampus (HPC) are temporarily attenuated through medial septum (MS) inactivation and replaced with electrical stimulation triggered by ongoing oscillations in the supramammillary area (SuM). This dataset has the advantage of being real brain local field potential (LFP) recordings capturing the full complexity of a biological signal. Also, our manipulation externally imposes “causality” of known directionality to test and compare different directed connectivity estimators (DCE). The nature of the circuit and our manipulations are outlined below.

HPC theta oscillations have various links to a plethora of behavioral processes (Buzsaki, 2005; Young, 2011). A large body of work indicates that the medial septum (MS) is crucial for the natural occurrence of theta oscillations in the HPC (Petsche and Stumpf, 1960; Bland, 1986; Buzsaki, 2002). Subcortical inputs to the MS through the posterior hypothalamic area (particularly the supramammillary nucleus; SuM) have been found to contribute critically to HPC theta oscillation control (Kirk and McNaughton, 1993; McNaughton et al., 1995; Thinschmidt et al., 1995). Since MS inactivation or lesion abolishes spontaneous HPC theta oscillations *in vivo* (Lawson and Bland, 1993), it is believed that SuM contributes to HPC theta exclusively through the MS despite its direct HPC innervations to the dentate gyrus and CA2 subregion (Vertes, 1992; Magloczky et al., 1994; Vertes and McKenna, 2000; Cui et al., 2013). Disynaptic projections from the HPC can return to SuM through the MS, the CA3 area via the lateral septum, or through subicular-hypothalamic backprojections (Shibata, 1987; Hayakawa et al., 1993; Kiss et al., 2002). However, it has been shown that SuM integrity is not always necessary for HPC theta to occur (Thinschmidt et al., 1995) and may merely reduce peak frequency in the freely moving rat (McNaughton et al., 1995). More recent data based on spike-field Granger causality have suggested a role for the SuM in modulating HPC theta at higher frequencies (Kocsis and Kaminski, 2006), which is related to water maze learning/performance (McNaughton et al., 2006; Ruan et al., 2011; Hernandez-Perez et al., 2015).

Based on our current understanding of the SuM-MS-HPC circuitry, we expect in rats swimming in the water maze that there will be a predominant HPC to SuM direction of theta control (Ruan et al., 2011). MS-inactivation should reduce the HPC outflow and favor a null or SuM to HPC direction of theta propagation. With irregular, non-periodic stimulation under MS-inactivation, HPC/SuM directed connectivity is expected to be comparable to MS-inactivation alone. HPC theta activities are expected to be driven by a periodic, stationary 7.7 Hz stimulus train delivered to the fornix (James et al., 1977), while MS is inactivated, and exerts its influence on the SuM relayed through the LS to favor a HPC to SuM direction of theta propagation. Finally, when HPC activity is replaced and driven rhythmically by peaks of ongoing SuM theta activity, we expect an unequivocal SuM to HPC direction of theta propagation.

The use of our HPC-SuM data is advantageous given: (1) the dominant interaction is known to occur in a single (theta) frequency band and; (2) it is a simple bivariate system that mitigates further complexity (e.g., common input, extensive multi-directional coupling) in multivariate systems. The goals of the current study are to: (1) use a unique biological dataset with known, unequivocal directionality/Granger causality to assess differences and similarities in various directed connectivity measures and; (2) assess the influence of factors known to affect signal processing in general, particularly directed connectivity, such as coupling strength, signal-to-noise ratio and stationarity.

## Methods

### Subjects

Twenty-three male Sprague-Dawley rats were included in this study. There is an incomplete overlap between the dataset used for the current analysis and data published previously (McNaughton et al., 2006; Ruan et al., 2011, 2017). The animals were obtained from the Department of Laboratory Animal Sciences, University of Otago and allowed to acclimatize to the laboratory for at least 10 days. After ketamine (75 mg/kg, 100 mg/ml, Parnell Laboratories New Zealand Limited) and medetomidine hydrochloride (0.5 mg/kg, 1 mg/ml, Novartis Animal Health Australia Limited) anesthesia, bipolar twisted wire (70 μm diameter) electrodes and a guide cannula (Plastics One Inc. Roanoke, VA 24022), were stereotaxically implanted using aseptic surgical procedures. HPC recording electrode was aimed at the CA1/dentate layers (AP −3.8 mm, ML −2.5 mm, DV −3.5 mm, tip separation 1.0 mm) and the SuM recording electrode (AP −4.8 mm, ML −0.9 mm, DV −9.4 mm, tip separation 0.5 mm, 6.0° from vertical) targeted the parvicellular supramammillary nucleus (Pan and McNaughton, 2004). A fornix stimulating electrode (AP −1.0 mm, ML −1.0 mm, DV −4.0 mm, tip separation 0.5 mm, 8.0° from vertical) and a 26 GA cannula guide at the medial septal area (AP +0.2 mm, M-L −1.04 mm, D −5.91 mm, 10.0° from vertical) were also implanted. An uninsulated silver ground wire wound around one of five anchor screws, along with all other electrodes were inserted into a McIntyre connector and secured with dental cement. Atipamezole (2.5 mg/kg, 5 mg/ml, Novartis Animal Health Australia Limited) was administered to assist recovery from anesthesia, followed by postoperative analgesic administration. A 10 day rest period was imposed before any recording and behavioral testing began. All protocols described here were approved by the University of Otago Animal Ethics Committee (84/00 and 67/03).

### Electrical recording

Recordings from the HPC and the SuM were fed to amplifiers (Grass P511K, 1–30 Hz band pass filter) through a custom-made source follower. The data were digitized at 100 Hz and acquired by a Micro1401 (CED, UK) using the Spike2 software. The LFPs from HPC and SuM were acquired for the whole behavioral session, starting prior to any MS injections or fornix stimulations, terminating once the effects of the drug and/or stimulation had worn off as judged qualitatively by the recovery of HPC theta amplitude.

### MS blockade and fornix stimulation

The rats were assigned to five groups: (1) control group (CON, *n* = 5) without any drug administration or stimulation; (2) animals receiving MS 2% tetracaine (0.5 μl; Sigma) only (TET, *n* = 4); (3) animals that received tetracaine and irregular (non-periodic, averaged 7.7 Hz) fornix stimulation (IRR, *n* = 4); (4) animals that received tetracaine and a regular/stationary, 7.7 Hz fornix stimulation (REG, *n* = 5) and; (5) animals that received tetracaine and a non-stationary oscillatory stimulus that was paced by the peak of the concurrently recorded SuM LFP (BP, *n* = 5). MS tetracaine was injected through a 33 GA injection cannula via a microsyringe pump (Razel Scientific Instruments, Inc., Stamford, CT) at 0.5 μl/min. The electrical stimuli were only applied to the fornix if a clear decrease of HPC LFP power and a loss of theta rhythmicity were observed.

In all stimulation conditions, a threshold 2–10 V/0.5 ms pulse was applied to the fornix (James et al., 1977). In the IRR condition, the stimulus was generated by a random number generator, which generated stimulus trains that were non-periodic, but had an averaged frequency of 7.7 Hz. In the REG condition, a stationary 7.7 Hz train was delivered continuously. In the BP condition, SuM LFPs were low-pass filtered with DC adjustment through a separate low-gain amplifier. This amplified signal was then fed into a stimulator by triggering it at the positive peak of each theta cycle. The stimulator was switched on after the trigger to SuM theta peak was satisfactory. The stimulation intensity was adjusted until the pulses were phase locked to the ongoing HPC LFP, and was continuously monitored and adjusted as necessary.

### Behavioral tasks

#### Water maze

A 1-day water maze training protocol was adopted in this study. A 150 cm wide, 35 cm deep black circular pool was filled with 26°C (±2°C) water. A black, 15 × 15 cm square platform was positioned 1.5 cm below the water surface in the middle of the southeast quadrant. Each trial consisted of releasing the rat facing the wall of the pool, allowing 60 s to locate the platform with a 15 s inter-trial interval, during which the animals stayed on the platform. The animals were guided to the platform if they failed to locate it. In the 16 trials administered, the point of entry to the pool was counterbalanced (NSWE ENSW WENS SWEN). Since we used a 1-day water maze learning protocol with a 15 s inter-trial interval, no control of body temperature loss was implemented. The behavioral data were acquired and collated by HVS (HVS Image Ltd., UK). A digital trigger was fed from the HVS hardware to Spike2 to mark the start and end of each trial.

#### Open field

Rats were exposed to a square box (73 × 73 × 51 cm) for 6 min.

#### Operant conditioning

Access to food was gradually limited to 1 h a day, after shaping/conditioning, over a 10 day period. Rats were shaped and trained on a continuous reinforcement schedule (CRF) until a stable performance (i.e., >100 lever presses for at least four consecutive days) was reached. Then, the rats were trained on a fixed-interval (FI) schedule where the first lever press initiates a 60 s interval between rewarded lever presses. All sessions lasted for 30 min. The last session of each schedule type (i.e., CRF or FI) was used for the current analysis.

### Histological reconstruction

At the end of the experiment, the rats were deeply anesthetized with sodium pentobarbital and transcardially perfused with physiological (0.9%) saline followed by 10% formalin in physiological saline. The brains were extracted for further fixation with 10% formalin for 24 h before cryoprotection with 30% sucrose. Upon saturation, brains were sectioned on a freezing microtome at 90 μm. Electrode and cannula locations were reconstructed to validate targeting of recording sites.

### Data pre-processing

The LPFs from WM sessions were segmented into single trials based on the digital triggers from the HVS hardware. Due to previously reported experience-dependent changes in HPC-SuM interactions in the water maze (Ruan et al., 2011), only the first five >2 s trials from each animal were included in this analysis to ensure consistency between datasets. Additional trials were excluded if the stimulation parameters were not optimized and required further adjustments (e.g., if simulation intensity was not consistent throughout the trials considered). After the removal of the direct component in the recordings, the segmented raw LFPs were exported to Matlab (Mathworks, Natwick, MA) for further analysis. The data segments from other conditions (OF, CRF and FI) were duration-matched to the WM segments to yield equal number of sessions/samples for comparison across conditions. Specifically, CRF data from the day rats reached criterion were used. For FI, the last available session with successful MS-inactivation and SuM-triggered HPC driving via the fornix was used. All data were z-scored for further analyses.

### Spectral analysis

Spectrograms and cohereograms were computed by the multi-taper method using the Chronux toolbox (Bokil et al., 2010). Three tapers with a band-width product of 5 were used to calculate the power and coherence spectra. The spectral estimates were made in 2 s bins with 90% overlap, using a periodogram method. Coherence was assessed as the normalized cross-spectra from the HPC and SuM.

Signal-to-noise ratio (SNR) was calculated as the ratio of theta band (5–12 Hz) power spectra density to the rest of the spectrum (0–50 Hz).

### Pair-wise phase consistency

Pair-wise phase consistency (PPC) was calculated to better reflect phase coupling alone between the HPC and SuM, as our manipulations affect both the amplitude and phase of recorded LFPs. Data were filtered (5–12 Hz) and Hilbert-transformed for phase extraction. Relative angular distances between phase angles from HPC and SuM were used to calculate the degree of phase coupling (Vinck et al., 2010).

### Granger causality

Methodologies described here follow the concept where *a* causes *b* if the past of *a* can serve as a good predictor of the present/future of *b* (Granger, 1969). In these approaches, z-scored LFPs are fitted with a step-wise least squares auto-regressive model with model orders determined by Bayesian information criterion (BIC). Max model order (*p*) to attempt fit was set at 20, with the optimal model order for most trials being 8 ± 4. As with spectral analyses described above, 2 s, 90% overlapping windows of data were submitted for further analysis. Multivariate auto-regressive (MVAR) model coefficients were then calculated with optimal model orders determined for each trial (i.e., all windows used the same model order derived from session model order estimation). The MVAR coefficients (*A*) were then transformed into the frequency domain (*f*) before further processing (eq. 1). Formulations and calculations are based on previous descriptions (Gourevitch et al., 2006; Cui et al., 2008; Young and Eggermont, 2009).

(1) $$\begin{array}{l} A\left(f\right)=\;\overset{p}{\underset{k=0}{\sum }}A_{k}e^{\left(i2\pi fk\right)} \end{array}$$

#### Partial directed coherence

For PDC (Baccala and Sameshima, 2001), the frequency-transformed MVAR coefficients are subtracted from the identity matrix (*A*_*ab*_*(f)*) and are normalized against the “out” flow (i.e., Hermitian transposed coefficients in dimension *a* × *b*, denoted as Q in Equation 2).

(2) $$\begin{array}{l} PDC_{ab}\left(f\right)=\;\frac{A_{ab}\left(f\right)}{\sqrt{\sum_{k=1}^{Q}{\left|A_{kb}\left(f\right)\right|}^{2}}} \end{array}$$

#### Directed transfer function

Given our application, the normalized, direct form of DTF is essentially mathematically equivalent to PDC, with the main difference being matrix inversion and normalization. In a bivariate system, the estimate of in/out “flow” of information is equivalent. Also, the ability for DTF to distinguish direct and indirect influences is irrelevant in a bivariate system. Therefore, we computed “raw” DTF (Kaminski and Blinowska, 1991), where a matrix inversion is applied to the frequency-transformed coefficients and no normalization was implemented (Equation 3).

(3) $$\begin{array}{l} DTF_{ab}\left(f\right)=\;|A_{ab}{\left(f\right)}^{-1}| \end{array}$$

#### Directed coherence

DCOH (Saito and Harashima, 1981) implemented here is a variation of the DTF (i.e., with matrix inversion, *H(f)* = *A(f)*^−1^) above, but with a randomly generated noised term (*c*) added to the MVAR model coefficients prior to frequency transformation, and, in our case, is normalized by the sum of cross-spectra from all possible combinations of HPC, SuM and the noise term.

(4) $$\begin{array}{l} DCOH_{ab}\left(f\right)=\;\frac{H_{ab}\left(f\right)}{\sqrt{{\left|H_{aa}\left(f\right)\right|}^{2}+{\left|H_{ac}\left(f\right)\right|}^{2}+{\left|H_{ab}\left(f\right)\right|}^{2}}} \end{array}$$

#### Geweke-Granger causality

Formulated according to the original conception of Granger causality (Geweke, 1982), the natural log of spectral power (*S*_*aa*_) from HPC is normalized against the intrinsic power (defined as the difference between channel HPC power (*S*_*aa*_) and the product of noise covariance (*C*_*bb*_ − $C_{ab}^{2}$*/C*_*aa*_) and transfer matrices derived from HPC and SuM; (*|H*_*af*_*|*^2^)) is taken as the estimate of SuM to HPC causal influence (Cui et al., 2008).

(5) $$\begin{array}{l} GGC_{ab}\left(f\right)=\ln\;\left(\frac{S_{aa}\left(f\right)}{S_{aa}\left(f\right)-\left(C_{bb}-\frac{C_{ab}^{2}}{C_{aa}}\right)\;{\left|H_{af}\left(f\right)\right|}^{2}}\right) \end{array}$$

### Non-MVAR directional estimates

#### Phase transfer entropy

Phase transfer entropy is essentially a natural extension of the use of transfer entropy for non-linear causal inference (Lobier et al., 2014). Band-passed phase angles (θ) obtained from Hilbert transform are binned to compute conditional probabilities for phases relating to lagged (*t*′) and no-lag (*t*) phase states. An estimate is based on how much information (*H*) can be predicted by one input over the other, taking into account phase distributions at different time lags (Equation 6).

(6) $$\begin{array}{l} PTE_{ab}=H\left(\theta_{b}\left(t\right),\theta_{b}\left(t^{\prime }\right)\right)+H\left(\theta_{b}\left(t^{\prime }\right),\theta_{a}\left(t^{\prime }\right)\right)\\ -\;H\left(\theta_{b}\left(t^{\prime }\right)\right)-H\left(\theta_{b}\left(t\right),\theta_{b}\left(t^{\prime }\right),\theta_{a}\left(t^{\prime }\right)\right) \end{array}$$

Specifically, we filtered input data at 0.5 Hz steps with 1 Hz bandwidth across the whole spectrum (i.e., 0.5–49.5 Hz) in forward and reverse to minimize phase distortions and computed PTE in defined narrowband inputs across time. As above, a 2 s, 90% overlapping window was used. We adopted the Freedman-Diaconis rule (Freedman and Diaconis, 1981) for the estimation of bin sizes for phase angles. Although we present PTE as a non-linear, phase-based measure of directed connectivity, it is clear that it is theoretically similar to Granger causality (Seghouane and Amari, 2012), as the determination of causal influence is dependent on time lags and changes to metric when taking into account the past of a different signal.

#### Phase-slope index

As its name suggests, this measure of directed connectivity involves the calculation of phase slopes, or specifically, the change of phase differences as a function of frequency (Equation 7).

(7) $$\begin{array}{l} PSI_{ab}=\;ℑ\left(\sum_{f\in F}C_{ab}^{*}\left(f\right)C_{ab}\left(f+\delta f\right)\right) \end{array}$$

This estimation is implemented by taking the imaginary part (ℑ) of spectral coherence (*C*) as estimates of phase differences within a frequency bandwidth (*F*), which was 1 Hz as in PTE, in 0.5 Hz steps across the whole spectrum. Given our (2 s) windowed application of PSI, we used the raw estimates instead of normalized version of PSI for further analysis, since normalization uses the standard deviation of phase slope differences (Nolte et al., 2008) and our data segments are short (2 s).

### Statistical analysis

#### Surrogate data

Given the goal of this study was to compare different methods as well as different experimental conditions, we decided establishing significance levels using a single approach for the entire dataset was appropriate. Instead of using phase randomization or time-reversal techniques, we opted to adopt a modified version of trial-shuffling to maximise “biological-ness” of our surrogate sets. Specifically, we scrambled HPC and SuM pairings from all available segments of 2 s data across all conditions to generate 20,000 unique, non-repeating pairs for surrogate data analysis. In our shuffling scheme, only the pairing is shuffled but not channel identity (i.e., HPC and SuM contained data from their respective labels). The choice of 20,000 segments is to match the number of segments in the experimental condition with the most data (17,653 segments). Finally, the DCE from the shuffled pairs were used to generate indices, as done for experimental data, by subtracting SuM to HPC influence from HPC to SuM influence. A 95% confidence interval was calculated from the distribution of these indices and served as thresholds for determining significant causal influences. Surrogate data sets were generated separately for comparing different experimental manipulations between groups, and for comparing different behavioral contexts for data collected from the same rats.

#### Analysis of variance

One-way ANOVAs were applied to determine statistical significance for spectral and non-directed connectivity differences between experimental groups (CON, TET, IRR, REG, and BP). Repeated-measures ANOVAs were conducted to examine the role of the behavioral context (operant responding: CRF and FI; ambulation; OF and WM) and the effect of stimulation (no stimulation and SuM theta LFP-triggered fornix stimulation under MS-inactivation). *Post-hoc* comparisons were corrected by using the Bonferroni method. Bonferroni corrected *p-*values and raw *p-*values that remain significant after Bonferroni correction are reported accordingly.

#### Correlation

Pearson's correlation coefficient and associated *p*-values are used to determine the correspondence of frequency and time domain representations between the different directed connectivity estimates. In addition, correlations and their statistical significance between connectivity measures and signal properties such as stationarity and SNR were calculated to estimate how these properties may bias connectivity measures overall, and/or the magnitude of estimates for either direction of causal influence. Steiger's test (Steiger, 1980) was used to assess whether dependent correlations, between signal properties (e.g., coupling strength) and directed connectivity (e.g., PDC), differ between HPC to SuM and SuM to HPC directions.

### Source of connectivity estimation bias

Signal-to-noise ratio (SNR), stationarity, volume conduction and common input are known to affect the accurate estimation of directed connectivity (Bastos and Schoffelen, 2015). Given that our recordings are bipolar, we expect volume conduction to be minimal. The issue of common input in the context of missing intervening nodes in our circuit is not direct addressable since we only recorded from the HPC and SuM. In this study, we explored how SNR and stationarity relate to DCEs.

#### SNR

The effects of SNR are explored in two contexts: (1) total SNR as the sum of HPC and SuM spectral power at theta range (5–12 Hz) over the whole power spectrum and; (2) SNR difference ratio as the difference between HPC and SuM theta SNR over the sum of HPC and SuM theta SNR. The former is used to estimate the dependence of DCEs on overall SNR, while the latter is used to gauge the effect of the unbalanced difference in theta power between the HPC and SuM (i.e., weak nodes).

#### Stationarity

As LFPs are routinely detrended prior to directed connectivity analyses (as we have done in our analyses), and since oscillatory LFPs are essentially mean-stationary with a stable direct component (Kipinski et al., 2011), we examined the role of variance-stationarity in its potential biasing of DCEs. Specifically, we calculated the heteroscedasticity of LFP segments using the White test (White, 1980). We used the Lagrange multiplier (LM) as the metric to represent variance-stationarity. A logarithmic transform was performed on LMs to approximate their distribution to a normal one. In addition, given our main interest is in oscillatory LFPs, we also assessed the periodicity of our LFP segments by computing the auto-correlation function, and the decay constant from fitting the peaks of the auto-correlation function across time lags (tau). Specifically, a five-point moving average of the auto-correlation function was log-transformed and fitted with linear regression to derive tau (slope coefficient) as an indicator of the exponential decay rate of the auto-correlation function. Tau was also log-transformed to approximate normality. As with SNR measures, since DCEs describe the relationship between the HPC and SuM, we summed the LM and tau metrics to provide a combined measure of variance and periodicity–stationarity, respectively.

## Results

**Figure 1:**
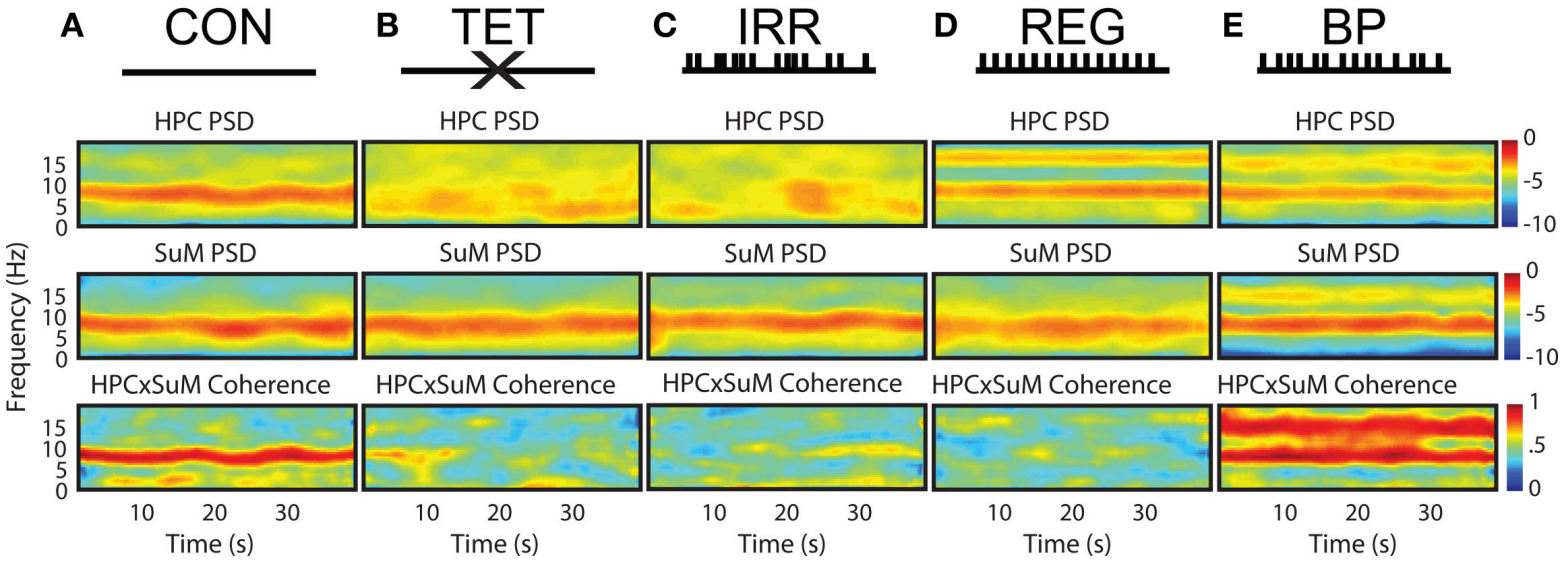
Summary of experimental manipulations and their time-frequency representations in examples of water maze swimming data. **(A)** In CON, with no external manipulations. HPC, SuM spectrograms and HPCxSuM cohereogram show high agreement in the power and frequency fluctuation through the session. **(B)** In the TET condition, there is a general loss of HPC theta power, while SuM theta PSD remain relatively intact. A drastic reduction of HPCxSuM theta coherence is observed. **(C)** In IRR, the pattern of little theta PSD, intact SuM theta PSD and a reduction of HPCxSuM theta coherence similar to TET was observed. **(D)** REG condition drove HPC theta activities and its first harmonic. SuM theta appear to be unaffected by the manipulation, while ordinary coherence between HPC and SuM remain low. **(E)** In BP, clear HPC and SuM theta and their first harmonic can be seen in the spectrograms, with HPCxSuM cohereogram mirroring high magnitude PSD at theta and its harmonic. CON, control; TET, tetracaine; IRR, irregular; REG, regular at 7.7 Hz; BP, by-pass; HPC, hippocampus; SuM, supramammillary nucleus; PSD, power spectral density.

Figure 1 outlines the experimental conditions and the spectral profiles from HPC and SuM recordings. Compared to the CON condition (Figure 1A), MS-inactivation severely attenuated HPC theta power (Figure 1B, HPC PSD) while leaving SuM theta power largely unaffected (Figure 1B, SuM PSD), resulting in low and sporadic theta coherence between the two structures (Figure 1B, HPCxSuM coherence). Irregular stimulation (Figure 1C) essentially reproduced the same spectral profile seen in the MS-inactivation condition (Figure 1B). Fornix stimulation with a stationary 7.7 Hz stimulus train resulted in highly stable 7.7 Hz theta power and its first harmonic in the HPC (Figure 1D, HPC PSD), with no observable effects in the SuM in this example (Figure 1D, SuM PSD), and no observable coherence above background at the theta range (Figure 1D, HPCxSuM coherence). In the BP condition, clear theta LFP and its first harmonic can be seen in the HPC and SuM, with activities at these frequencies being highly coherent (Figure 1E).

### Non-directed connectivity

**Figure 2:**
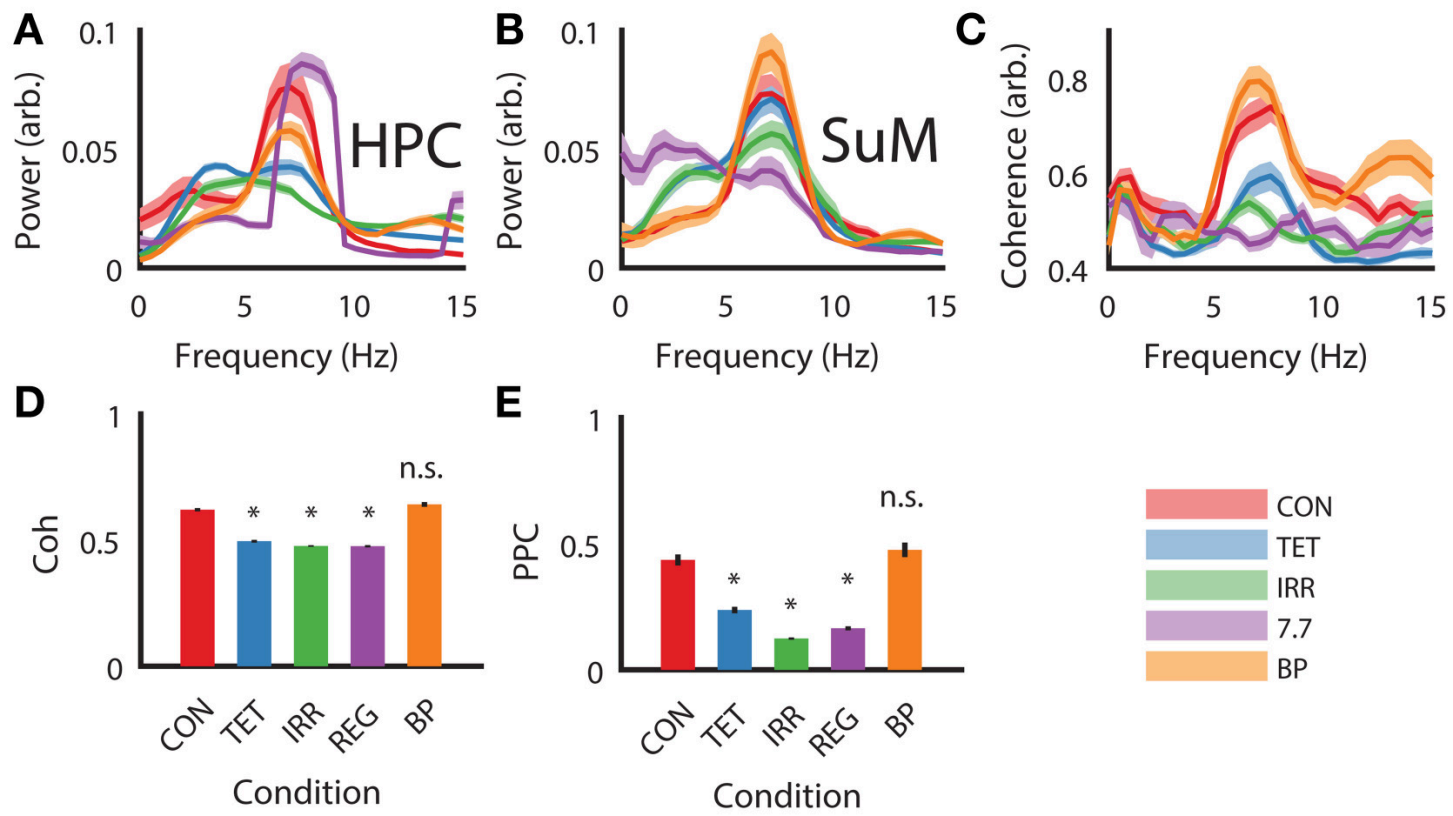
Power spectral density, coherence and PPC comparisons across different experimental conditions. **(A)** Group HPC PSD mirrors time-frequency representation presented in Figure 1—theta power is comparable between CON, REG, and BP conditions and higher than TET and IRR conditions, with REG theta frequency shifting to imposed 7.7 Hz. **(B)** Group SuM PSD showed minimal changes in PSD across the conditions, with the exception of REG condition, where theta power was greatly attenuated and slower oscillations predominate. **(C)** Theta coherence magnitude is similar between BP and CON, but low for TET, IRR and REG, with the latter having the lowest coherence. **(D)** Averaged theta band (5–12 Hz) coherence as in **(C)**. **(E)** Averaged theta (5–12 Hz) PPC differences across conditions are consistent with ordinary coherences but have a larger dynamic range. Asterisks indicate statistical significant, *post-hoc* differences compared to the CON condition, *p* < 0.001. CON, control; TET, tetracaine; IRR, irregular; REG, regular at 7.7 Hz; BP, by-pass; HPC, hippocampus; SuM, supramammillary nucleus; PSD, power spectral density; PPC, pairwise-phase consistency.

As expected from our manipulations and reported previously (McNaughton et al., 2006), strong theta oscillations occurred during CON, BP, and REG conditions and were attenuated during TET and IRR conditions in the HPC (Figure 2A). Qualitatively, the peak of the REG condition is centered at the stimulation frequency of 7.7 Hz, higher than the CON and BP conditions, which have comparable center frequencies at 7 Hz. Theta power was comparable between the CON and REG conditions (*p* = 0.59, Bonferroni corrected), whereas all other conditions had lower theta power compared to CON (*p* < 0.002, Bonferroni corrected).

MS-inactivation and fornix stimulation had a significant [*F*_(4, 98)_ = 7.88, *p* < 0.001] but different impact on the SuM compared to HPC (Figure 2B). Instead, theta power appeared the highest in the BP condition, followed by CON, TET and IRR conditions, with no statistically significant difference in theta power comparing these conditions. The center frequency within 5–12 Hz is also qualitatively invariant across conditions at ~7 Hz. Interestingly, SuM theta power remained the lowest during the REG condition compared to CON (*p* < 0.001, Bonferroni corrected). We note delta (2–4 Hz) oscillations are prominent in the SuM under TET, IRR, and REG conditions.

Theta coherence between the HPC and SuM were the highest in CON and BP conditions and are low for TET, IRR and REG conditions [*F*_(4, 98)_ = 33.30, *p* < 0.001; Figure 2C]. Particularly, theta coherence is significantly reduced in all conditions where MS was inactivated (*p* < 0.001, Bonferroni corrected), except for the BP condition where theta coherence was found to be virtually identical to CON (Figures 2C,D; *p* > 0.999, Bonferroni corrected). As indicated in Figure 1E, there is also a high level of coherence at the first theta harmonic range in the BP condition (Figure 2C, orange line at ~13 Hz). PPC measures are in good agreement with coherence measures (Figure 2E), with our manipulation producing changes in phase coupling [*F*_(4, 98)_ = 17.46, *p* < 0.001]. Again, phase coupling at theta range in TET, IRR and REG conditions were all attenuated (*p* < 0.001, Bonferroni corrected) compared to CON, while remaining high for the BP group (*p* > 0.999, Bonferroni corrected).

### Directed connectivity

**Figure 3:**
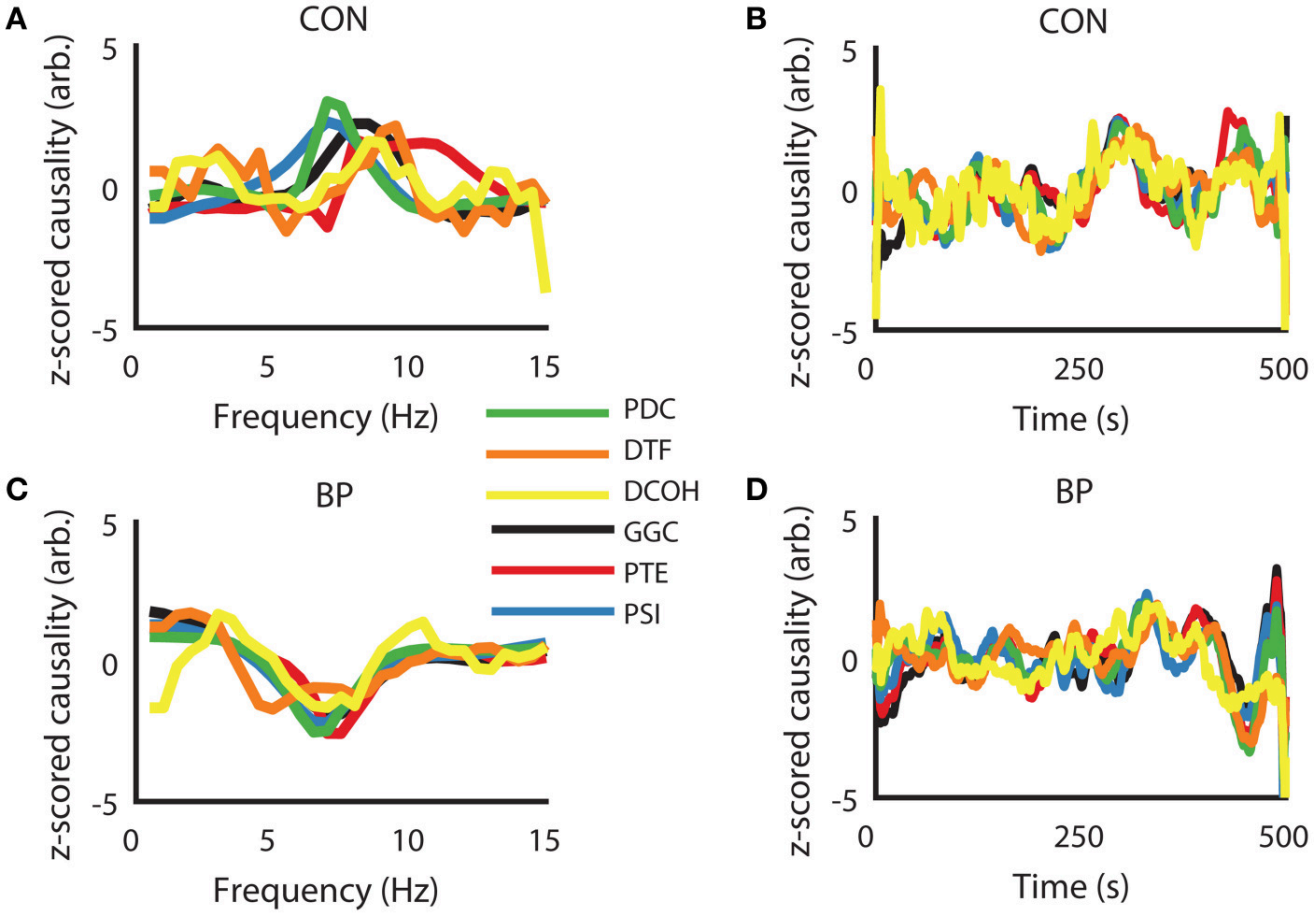
Examples of similarities in frequency- and time-representations across different directed connectivity estimates shown as z-scored difference scores. **(A)** In the CON condition, all estimators returned a peak within our frequency band of interest (5–12 Hz), with variability in peak frequency and the magnitude at each frequency across the methods of estimation. **(B)** In the time domain, there is general agreement at slower timescales but relatively higher variability at fast timescales. **(C)** Under BP condition, frequency representations across all estimators are highly congruent, with peak frequency and the widths of the “theta peak” showing much better agreement compared to CON condition. **(D)** Time-domain agreement under the BP condition appears to be qualitatively better than the CON condition. CON, control; BP, by-pass; PDC, partial directed coherence; DTF, directed transfer function; DCOH, directed coherence; GGC, Geweke-Granger causality; PTE, phase transfer function; PSI, phase slope index.

To visually present the frequency domain representation of each DCEs, we computed DCEs from the full spectrum (0–50 Hz) for each measure, took the z-scored difference between HPC to SuM and the reciprocal estimates to visualize their differences in the frequency domain (raw data were used for further analysis). Using the CON condition as an example, where bi-directional influences are expected, a positive peak in and around our defined theta range (5–12 Hz) indicates a predominant HPC to SuM direction of communication. However, there appears to be little correspondence between measures, with PDC, GGC, and DCOH having smoother spectra frequency domain representation (Figure 3A). The fluctuations of all the DCEs in the time domain (i.e., averaged 5–12 Hz values for each estimate across time) appear to have better correlation with each other at a longer timescale than short (Figure 3B). In contrast, all DCEs in the BP condition are in better general agreement compared to CON, with less variability in the (negative) peak position (at ~7 Hz) to indicate a predominantly SuM to HPC direction of directed influence (Figure 3C). Likewise, fluctuations in DCEs in the time domain under BP are also better correlated with each other compared to CON, as expected by the closed-loop nature of BP condition (Figure 3D).

**Figure 4:**
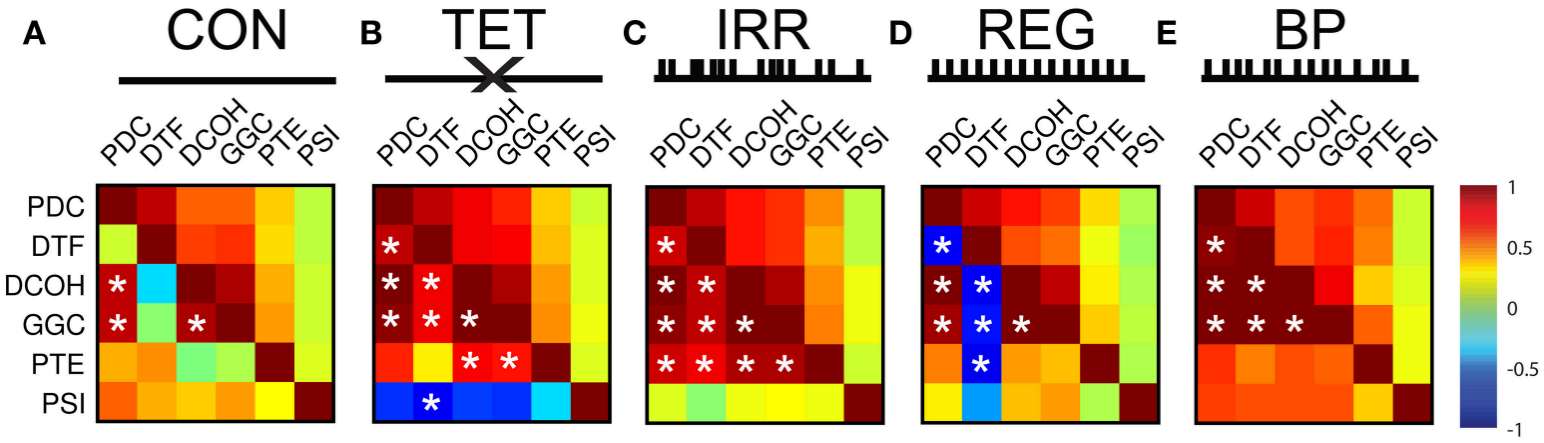
Correlation matrices for frequency- and time-representation similarities across different experimental conditions and different DCEs. For each matrix, the upper half represents correlation coefficients in the time domain (which are all statistically significant at *p* < 0.001). The lower half of the matrix represents correlation coefficients in the frequency domain, and statistically significant correlations after Bonferroni correction are marked with white asterisks (*p* < 0.05, Bonferroni corrected). **(A)** In CON, time-domain correlations are better among the MVAR-based methods, and there is no PSI correlation to other estimators. DTF is poorly correlated with other in the frequency domain. **(B)** In TET, there is increase time- and frequency-domain correspondence. PSI shares negative correlations with all other estimators. **(C)** In IRR, similar pattern of time- and frequency-domain correlations, with PSI showing little correlation with other estimators. **(D)** In REG, DTF frequency-domain representation anti-correlates with all other estimators. PSI frequency-domain correspondence is highly variable in REG. **(E)** In BP, highest frequency-domain correlations are seen, while PSI remains to be uncoupled in the time domain. CON, control; TET, tetracaine; IRR, irregular; REG, regular at 7.7 Hz; BP, by-pass; DCE, directed connectivity estimate; MVAR, multivariate auto-regressive modeling; DTF, directed transfer function; PSI, phase slope index.

To further quantify and explore the differences in DCE frequency and time domain representations, we calculated the correlation coefficients and associated *p*-values between all DCEs in the frequency and time domains (Figure 4). Each matrix in Figure 4 is presented so the upper half of the triangular matrix represents the correlation values in the time domain, and the lower half of the triangular matrix represents the correlation values in the frequency domain. All time-domain correlations were statistically significant after Bonferroni corrections (i.e., uncorrected *p* < 0.0001 for all correlations). In the CON condition (Figure 4A), there is less agreement between all the estimates in general compared to other conditions, although PDC, DCOH, and GGC share the highest correlations that were found to be statistically significant (uncorrected *p* < 0.001). Higher correlations are found in the time domain among the MVAR-based methods but not with non-MVAR methods. PSI shares little temporal correlation with other estimators. With MS-inactivation, there is an increased agreement among PDC, DCOH and GGC, as well as PTE (Figure 4B). However, PSI estimates show statistically non-significant negative correlations with all other measures in this condition. The general correspondence in the time domain remains largely the same as in CON. In the IRR condition (Figure 4C), the general observation made for TET condition holds, with PTE estimates now showing higher correlations to MVAR-based methods. A lack of correlation of PSI representation in the frequency and time domains compared to other estimators persist. Interestingly, for the REG condition (Figure 4D), DTF frequency representation became anti-correlated with all other measures, yielding statistically significant negative correlations (uncorrected, *p* < 0.001) except for PSI. PSI remains largely uncorrelated with other measures in the frequency and time domains. Lastly, in BP where SuM LFPs were used to drive HPC LFPs, there is close agreement among the MVAR-based methods, with PTE and PSI also sharing positive correlations with MVAR-based methods (Figure 4E). In sum, our BP manipulation, where SuM theta oscillations were imposed on the HPC through fornix stimulation, resulted in statistically significant and high correlation coefficients across all directed MVAR-based estimators.

**Figure 5:**
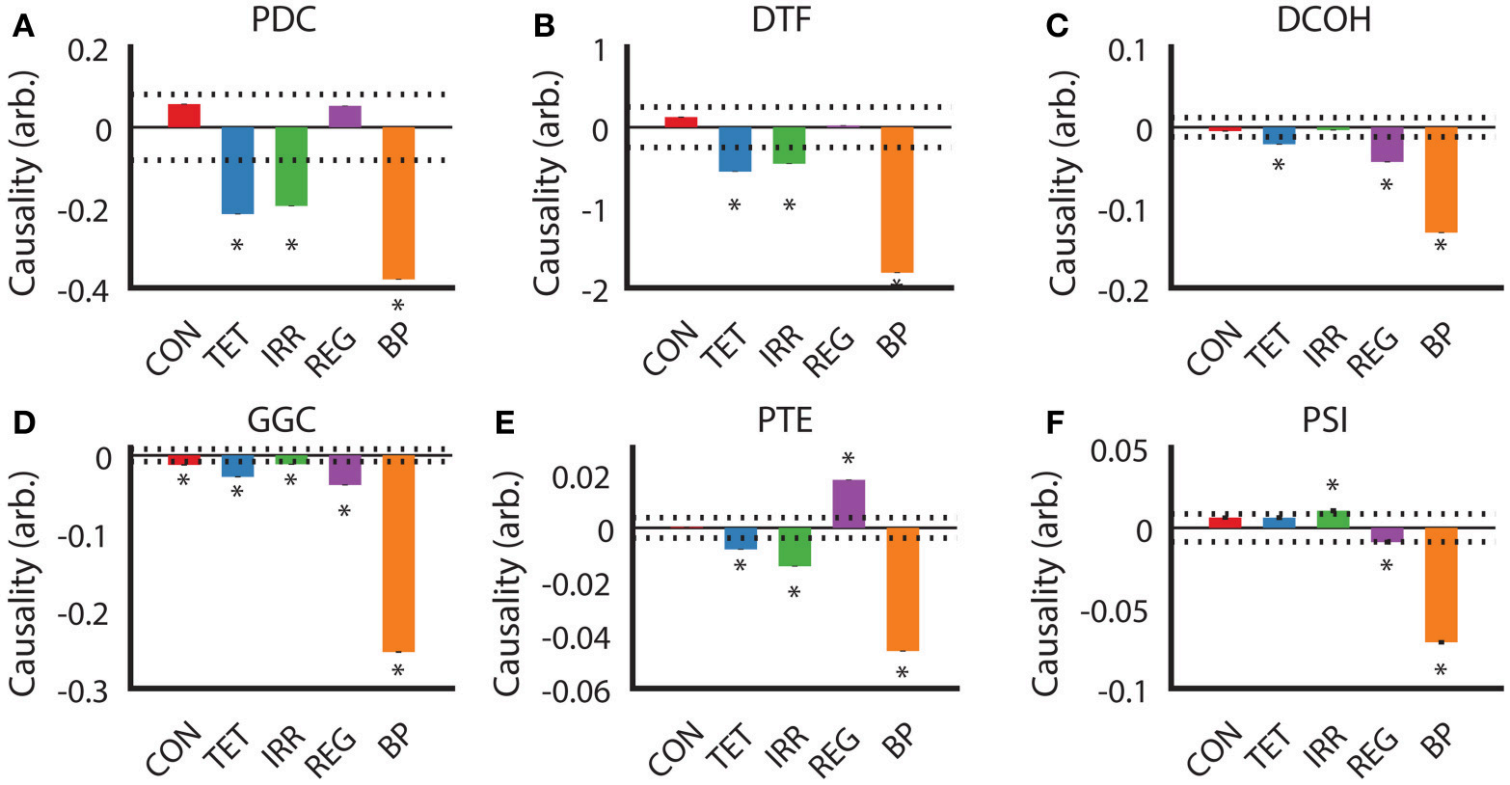
Determination of causal influences by directed connectivity estimators across experimental conditions. **(A)** PDC indicates under TET, IRR, and BP conditions, there is a statistically significant predominant SuM to HPC direction of causal influence. **(B)** DTF retuned the same pattern as PDC. **(C)** For DCOH, TET, REG, and BP all point to a statistically significant predominant SuM to HPC direction of causal influence. **(D)** All conditions have a statistically significant predominant SuM to HPC direction of causal influence assessed by GGC. **(E)** PTE revealed a predominant HPC to SuM causal influence in the REG condition, while TET, IRR, and BP causal influences are predominantly in the opposite direction. **(F)** PSI detected a marginal significant HPC to SuM causal influence in the IRR condition and an equally marginal influence in the opposite direction for REG. A predominant SuM to HPC causal influence was detected under PSI. Asterisks indicate mean values exceeding the 95% confidence interval drawn from a null distribution from trial-shuffled data. CON, control; TET, tetracaine; IRR, irregular; REG, regular at 7.7 Hz; BP, by-pass; HPC, hippocampus; SuM, supramammillary nucleus; PDC, partial directed coherence; DTF, directed transfer function; DCOH, directed coherence; GGC, Geweke-Granger causality; PTE, phase transfer function; PSI, phase slope index.

After establishing the similarities and differences between different DCEs across our experimental conditions, we examined the direction of estimated causality from each DCE across the conditions. For PDC and DTF estimates (Figures 5A,B), the direction, relative magnitude and statistically significant results are virtually identical, with only estimates with SuM to HPC causal influence from TET, IRR, and BP being statistically significant. Both CON and REG returned statistically non-significant HPC to SuM flow. As for DCOH, SuM to HPC causal influence was statistically significant in TET, REG, and BP conditions (Figure 5C). Causal influence from the SuM to HPC was uniformly statistically significant in the SuM to HPC direction across all conditions as assessed by GGC (Figure 5D). For PTE, TET, IRR, and BP conditions were all determined to have a statistically significant SuM to HPC flow, while REG had statistically significant flow in the opposite direction (Figure 5E). In disagreement with all other directed connectivity estimators, PSI returned a statistically significant HPC to SuM causal flow in IRR (Figure 5F), but detected significant SuM to HPC direction of causal influences that were detected in REG and BP conditions.

**Figure 6:**
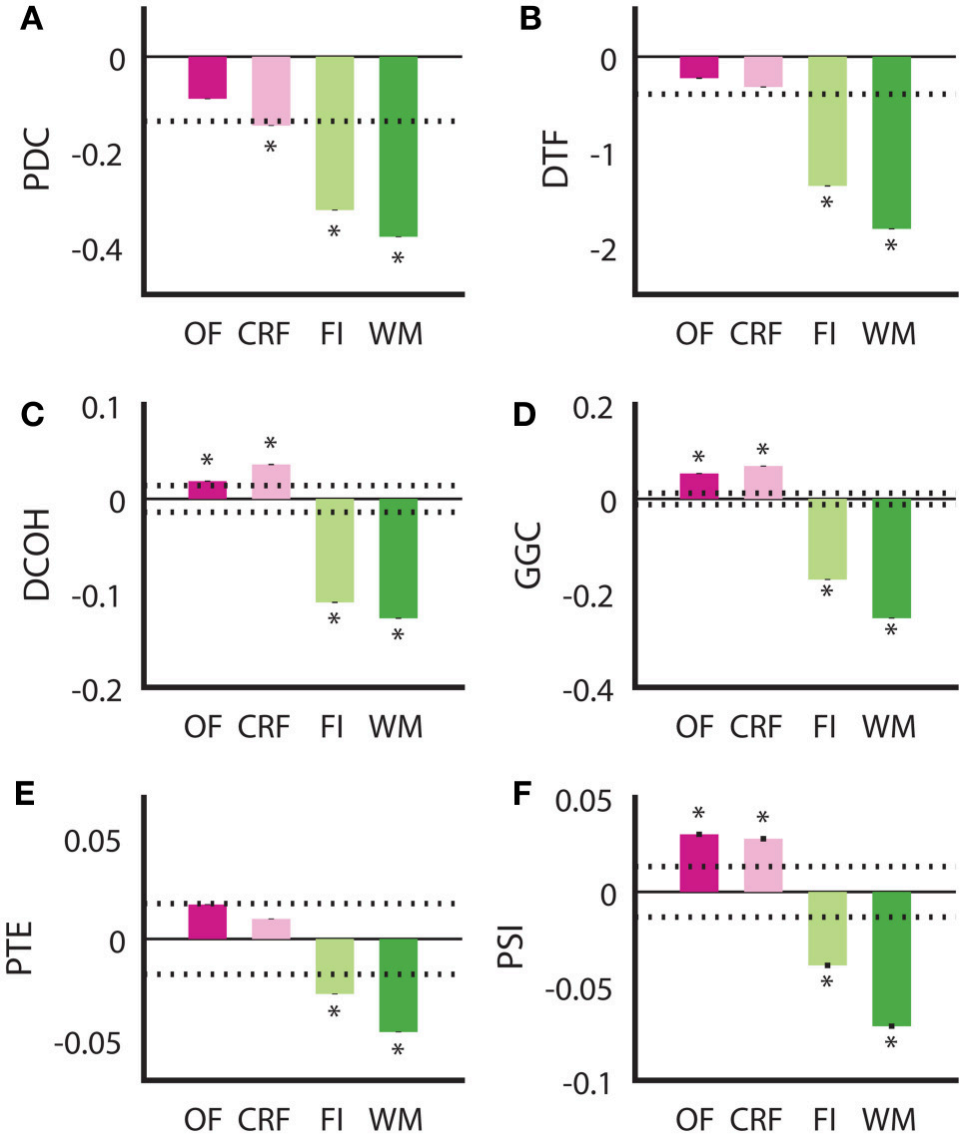
Examining consistency of directed connectivity estimates in the same rats placed in different behavioral and stimulation contexts. **(A)** In PDC, SuM to HPC direction of causal influences was detected in all behavioral contexts, but only significantly for CRF, FI, and WM. **(B)** DTF showed a similar pattern to PDC, but the averaged difference score in CRF did not reach significance. **(C)** DCOH detected a significant predominant HPC to SuM causal influence in OF and CRF but a significant predominant SuM to HPC causal influence in FI and WM. **(D)** GGC results mirror those computed by DCOH. **(E)** PTE also detected HPC to SuM direction of causal influence as DCOH and GGC, but these were not statistically significant. SuM to HPC causal influences for FI and WM are statistically significant. **(F)** PSI estimates returned the same pattern as DCOH and GGC, with a significant predominant HPC to SuM causal influence in OF and CRF, and a significant predominant SuM to HPC causal influence in FI and WM. Asterisks indicate mean values exceeding the 95% confidence interval drawn from a null distribution from trial-shuffled data. OF, open field; CRF, continuous reinforcement; FI, fixed interval; WM, water maze; PDC, partial directed coherence; DTF, directed transfer function; DCOH, directed coherence; GGC, Geweke-Granger causality; PTE, phase transfer function; PSI, phase slope index; HPC, hippocampus; SuM, supramammillary nucleus.

Although all DCEs were able to correctly determine the putative direction of theta modulation between the HPC and the SuM in the BP condition, none of the techniques were in complete agreement, or fully recapitulated the hypothesized directed connectivity based on our current understanding of the SuM-MS-HPC circuit outlined in our section Introduction. Our between-subjects design to compare the effect of MS-inactivation and fornix stimulation across the conditions may have been influenced by variability at the individual level between different groups, in addition to our manipulations. To ameliorate potential between-subjects bias, we compared the ability for each technique to differentiate no manipulation in the open field and in the operant chamber on a continuous reinforcement schedule (OF and CRF, respectively), to the closed-loop manipulation conditions in the water maze and fixed-interval schedule (WM and FI) in the same rats (*n* = 4). Essentially, the OF and CRF conditions served as “control” conditions where no manipulations took place, while WM and FI were treated as “experimental” conditions with MS-inactivation and fornix stimulation triggered by ongoing SuM theta LFPs. We also treated “operant responding” (CRF and FI) as a separate behavioral context from “ambulation” (OF and WM), As summarized in Figure 6, all methods are in agreement for the two conditions with our manipulation (FI and WM) indicated SuM to HPC direction of theta propagation [*F*_(1, 13, 672)_ = 323.27, *p* < 0.001]. However, there was no interaction between stimulation/no stimulation, and behavioral context [i.e., CRF/FI vs. OF/WM; *F*_(1, 13, 672)_ = 0.636, *p* = 0.653]. The differences between the different approaches can be distinguished into two groups: (1) PDC (Figure 6A) and DTF (Figure 6B), where a predominant SuM to HPC causality is seen for all conditions and; (2) DCOH (Figure 6C), GGC (Figure 6D), PTE (Figure 6E) and PSI (Figure 6F) where a HPC to SuM direction of causal influence is seen for OF and CRF conditions. Apart from difference in sign and magnitude of directed connectivity estimates, there are also differences in which condition yielded statistically significant results based on 95% confidence intervals generated by surrogate data. Specifically, under stimulation (imposing SuM theta oscillations onto HPC) conditions, all DCEs were statistically significant. Statistically significant HPC to SuM causal flow is detected by DCOH, GGC, and PSI in OF and CRF. PDC detects statistically significant SuM to HPC causal flow under CRF.

### Relationships between functional and directed connectivity

In theory, strong directed connectivity should be dependent on strong non-directed connectivity, given how coherence (real or imaginary) is an essential part of their computation. Here we compare how non-directed connection strength (i.e., coupling strength), as indicated by ordinary coherence and PPC, may affect directed connectivity measures.

**Figure 7:**
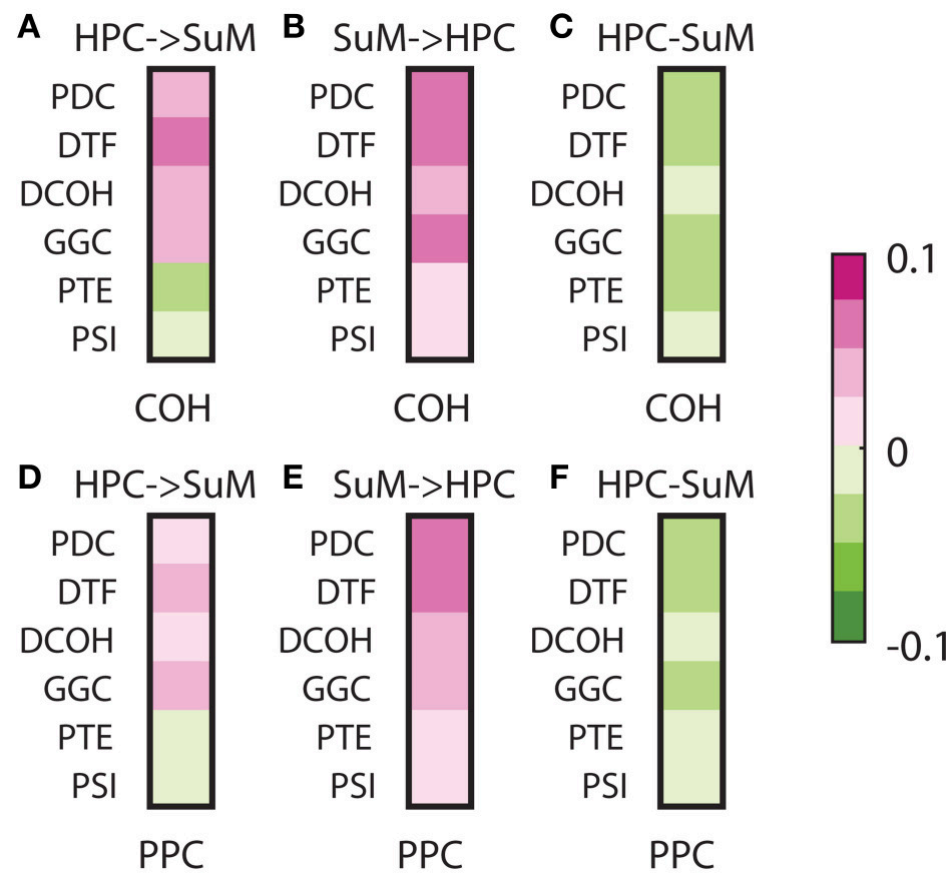
Correlations between non-directed and directed connectivity estimates. **(A)** Correlations between HPC to SuM estimates for each directed connectivity estimator and coherence (5–12 Hz). Most show positive correlations except for PTE and PSI. **(B)** Correlations between SuM to HPC estimates for each directed connectivity estimator and coherence (5–12 Hz). All show positive correlations. **(C)** Correlations between HPC-SuM difference score estimates for each directed connectivity estimator and coherence (5–12 Hz). DCOH and PSI appear to be least correlated with theta coherence. **(D)** Correlations between HPC to SuM estimates for each directed connectivity estimator and PPC (5–12 Hz) show similar pattern to those observed for coherence. **(E)** Correlations between SuM to HPC estimates for each directed connectivity estimator and PPC (5–12 Hz). All show positive correlations. **(F)** Correlations between HPC-SuM difference score estimates for each directed connectivity estimator and PPC (5–12 Hz). DCOH, PTE and PSI appear to be least correlated with PPC. DCOH, directed coherence; PTE, phase transfer function; PSI, phase slope index; HPC, hippocampus; SuM, supramammillary nucleus.

To assess the relationships between non-directed and directed connectivity, we correlated theta (5–12 Hz) coherence and PPC to directed connectivity at the same frequencies (Figure 7). In general, the relationship between coherence and PPC with directed connectivity measures is higher for the SuM to HPC direction than the reverse for all estimates except for PSI (see Table 1 for a summary of statistics). Given PSI directional estimates are essentially symmetrical for SuM to HPC and the reverse; no differences exist for PSI correlates to coherence and PPC. In sum, DCOH (*r* = −0.1591, *p* < 0.001 for coherence, *r* = −0.1056, *p* < 0.001 for PPC) and PSI (*r* = −0.0762, *p* < 0.001 for coherence, *r* = −0.0254, *p* < 0.001 for PPC) are two directed connectivity measures that show the lowest correlation with coupling strength, as measured by coherence and PPC (Figures 7C,F). PTE is relatively more coupled to coherence (*r* = −0.3228, *p* < 0.001) than PPC (*r* = −0.2164, *p* < 0.001). PDC (*r* = −0.4794, *p* < 0.001 for coherence, *r* = −0.3911, *p* < 0.001 for PPC), GGC (*r* = −0.3792, *p* < 0.001 for coherence, *r* = −0.2805, *p* < 0.001 for PPC) and DTF (*r* = −0.3300, *p* < 0.001 for coherence, *r* = −0.3170, *p* < 0.001 for PPC) are DCEs that show the highest significant correlations with coherence and PPC.

**Table 1:** Steiger's statistics and associated *p*-values for difference of dependent correlations.

**Directed**	**Undirected**	***Z***	***p*-value**
PDC	COH	54.236	<0.001
	PPC	41.297	<0.001
DTF	COH	13.457	<0.001
	PPC	16.412	<0.001
DCOH	COH	19.223	<0.001
	PPC	12.152	<0.001
GGC	COH	30.238	<0.001
	PPC	20.461	<0.001
PTE	COH	19.606	<0.001
	PPC	18.982	<0.001
PSI	COH	0	1
	PPC	0	1

### Signal-to-noise ratio, stationarity and directed connectivity

**Figure 8:**
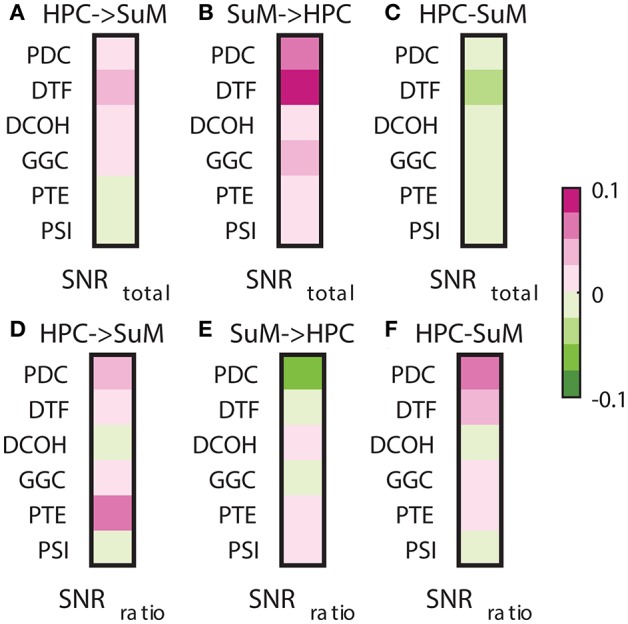
Correlations between SNR and directed connectivity estimates. **(A)** Correlations between HPC to SuM estimates for each directed connectivity estimator and total SNR. Most show positive correlations except for PTE and PSI. **(B)** Correlations between SuM to HPC estimates for each directed connectivity estimator and total SNR. All show positive correlations. **(C)** Correlations between HPC-SuM difference score estimates for each directed connectivity estimator and total SNR. DTF appears to be most affected by total SNR. **(D)** Correlations between HPC to SuM estimates for each directed connectivity estimator and SNR difference ratio. DCOH and PSI are least correlated to SNR difference ratio. **(E)** Correlations between SuM to HPC estimates for each directed connectivity estimator and SNR difference ratio. All but PTE switched sign of the correlation coefficients. **(F)** Correlations between HPC-SuM difference score estimates for each directed connectivity estimator and SNR difference ratio. PDC and DTF appear to be most affected by total SNR. PDC, partial directed coherence; DTF, directed transfer function; DCOH, directed coherence; PTE, phase transfer function; PSI, phase slope index; HPC, hippocampus; SuM, supramammillary nucleus.

Signal-to-noise ratio is an important aspect of signal processing in general, be it for spectral estimation, functional or DCEs. Here, we performed two tests based on the SNR of HPC and SuM theta power. First, we examined the relationship between combined SNR and DCEs. Total SNR appears to be better correlated with imposed SuM to HPC directionality (Figures 8A,B) in PDC (*Z* = 3.81, *p* < 0.001), DTF (*Z* = 3.19, *p* < 0.002) and GGC (*Z* = 3.60, *p* < 0.001), but do not differ for DCOH (*Z* = 1.68, *p* = 0.093), PTE (*Z* = 0.82, *p* = 0.421) and PSI (*Z* = 0, *p* = 1). Following the same trend, the lowest correlations between the directed connectivity difference scores (Figure 8C) are from DCOH (*r* = −0.0038, *p* < 0.001) and PSI (*r* = −0.0005, *p* < 0.001), suggesting these two methods are least affected by the total SNR of input data (Figure 8C). Conversely, DTF (*r* = −0.0306, *p* < 0.001) had the highest correlation, followed by PTE (*r* = −0.0271, *p* < 0.001), PDC (*r* = −0.0229, *p* < 0.001) and GGC (*r* = −0.0220, *p* < 0.001). In general, we report a weak relationship between total SNR and directed connectivity measures, where DTF is worst affected.

Next, we examined the relationship between the SNR difference ratio (as a measure of relative SNR between HPC and SuM) and directed connectivity (Figures 8D–F). SNR ratio did not correlate preferentially to directed connectivity estimates in either direction except for PTE (*Z* = 5.07, *p* < 0.001), where a higher correlation to SNR difference ratio was detected with HPC to SuM estimates compared to the reverse direction with no sign inversion (i.e., both correlations are positive). SNR difference ratio correlations are higher for SuM to HPC estimates compared to HPC to SuM estimates in PDC (*Z* = 2.28, *p* = 0.02), DTF (*Z* = 1.39, *p* = 0.16) and GGC (*Z* = 0.46, *p* = 0.64). Similar to total SNR, DCOH (*Z* = 0.15, *p* = 0.88) and PSI (*Z* = 0, *p* = 1) correlations to SNR difference ratio are not dependent on the direction of coupling. PDC (*r* = 0.0730, *p* < 0.001), DTF (*r* = 0.0278, *p* < 0.001) and PTE (*r* = 0.0212, *p* < 0.001) show highest correlations with SNR ratio, suggesting an unequal input SNR (i.e., presence of “weak” nodes) affects these estimates more than DCOH (*r* = −0.0007, *p* < 0.001), GGC (*r* = 0.0052, *p* < 0.001) and PSI (*r* = −0.0001, *p* = 0.80), which show near-zero correlations with SNR ratio (Figure 8F).

**Figure 9:**
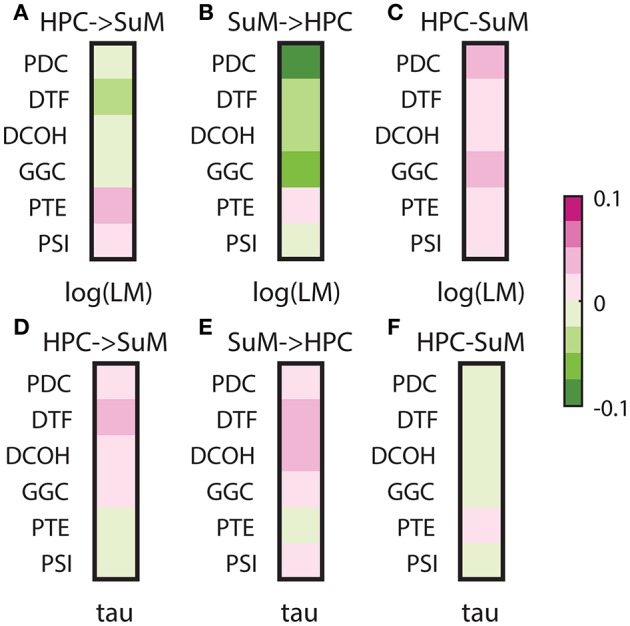
Correlations between stationarity and directed connectivity estimates. **(A)** Correlations between HPC and SuM estimates for each directed connectivity estimator and log-transformed Lagrange multiplier (LM). Negative correlations indicate a positive relationship between the magnitude of directed connectivity estimates and the heteroskedasticity of input data. MVAR-based methods weakly correlate to log-transformed LM, while PTE and PSI are positive correlated. **(B)** Correlations between SuM to HPC estimates for each directed connectivity estimator and log-transformed LM. All MVAR-based methods returned estimates highly correlated to heteroskedasticity. **(C)** Correlations between HPC-SuM difference score estimates for each directed connectivity estimator and log-transformed LM. PDC and GGC show higher correlation coefficients to log-transformed LM. **(D)** Correlations between HPC to SuM estimates for each directed connectivity estimator and tau, a measure of periodicity derived from decay constant of the auto-correlation function from input data. MVAR-based methods are positively correlated while PTE and PSI are weakly negatively correlated. **(E)** Correlations between SuM to HPC estimates for each directed connectivity estimator and periodicity. A pattern similar to **(D)** was observed, except for the switching of sign in PSI. **(F)** Correlations between HPC-SuM difference score estimates for each directed connectivity estimator and periodicity. All difference score estimates weakly and negatively correlate to periodicity, except for PTE, where the weak correlation is positive. LM, Lagrange multiplier; PDC, partial directed coherence; GGC, Geweke-Granger causality; PTE, phase transfer function; PSI, phase slope index; HPC, hippocampus; SuM, supramammillary nucleus.

Signal stationarity is essentially assumed in brain signal processing, given most algorithms depend on stationarity to yield correct outputs. It is widely recognized that brain signals are not stationary, but opinions differ as to if, and for how long an epoch, EEG/LFP signals may be considered stationary (McEwen and Anderson, 1975; Kawabata, 1976; Cohen and Sances, 1977; Kaplan et al., 2005; Kipinski et al., 2011). As discussed, LFPs are essentially mean-stationary but not variance-stationary; therefore, we tested the relationship between the degree of heteroscedasticity and the direction/magnitude of directed connectivity estimates (Figures 9A–C). All MVAR-based methods yielded negative correlations between either HPC to SuM or SuM to HPC directed connectivity estimates and log-transformed LM, essentially indicating lower magnitude of directed connectivity estimates are associated with more variance non-stationarity (Figures 9A,B). Only with PDC (*Z* = 6.63, *p* < 0.001) and GGC (*Z* = 4.83, *p* < 0.001) SuM to HPC correlations were higher than HPC to SuM, while difference in correlation was marginal in DCOH (*Z* = 2.29, *p* = 0.02) and non-significant for DTF (*Z* = 1.63, *p* = 0.10). Interestingly, although direction-dependent correlations were different for PTE (*Z* = 3.76, *p* < 0.001), in both cases the correlation was positive, suggesting higher variance non-stationarity is associated with higher magnitude PTE scores. PDC (*r* = 0.0451, *p* < 0.001) and GGC (*r* = 0.0299, *p* < 0.001) difference scores also showed highest correlation with heteroscedasticity, which indicate higher magnitude directed connectivity estimates are related to lower variance non-stationarity (Figure 9C). DTF (*r* = 0.0163, *p* < 0.001) and PTE (*r* = 0.0160, *p* < 0.001) difference scores shared similar but lower correlation with heteroscedasticity, followed by DCOH (*r* = 0.0058, *p* < 0.001). PSI (*r* = 0.0001, *p* = 0.13) had the lowest correlation with heteroscedasticity and the correlation was statistically non-significant (Figure 9C).

Lastly, given our signal of interest is theta oscillations between 5 and 12 Hz, we also examined how signal stationarity, in the context of periodicity, relates to directed connectivity measures (Figures 9D–F). Our data indicate there is no difference between HPC to SuM and SuM to HPC correlations to periodicity (all HPC to SuM vs. periodicity and SuM to HPC vs. periodicity comparisons, *p* > 0.32). All MVAR-based methods demonstrated positive correlations with periodicity, suggesting higher magnitude of directed connectivity estimates in either direction is related to more periodic inputs. However, PTE displayed the opposite trend, where PTE estimates in either direction were negatively correlated to periodicity. There is also essentially no relationship between the periodicity and directed connectivity difference scores, with Pearson's *r* approximating zero for MVAR-based methods (PDC, *r* = −0.002, *p* < 0.001; DTF, *r* = −0.0066 *p* < 0.001; DCOH, *r* = −0.0003 *p* = 0.03; and GGC *r* = −0.0010 *p* < 0.001) and/or lack any statistical significance in others (PTE, *r* = 0.0001, *p* = 0.06 and PSI, *r* = 0.0002, *p* < 0.001; Figure 9F). Collectively, periodicity appears to have minimal influence over DCEs.

## Discussion

In this study, we used a unique dataset where directional influence was artificially imposed via electrical stimulation in freely behaving rats. This dataset allowed us to use biological data to assess the performance of various functional and directed connectivity measures, instead of using simulated data that may closely approximate, but never fully recapitulate, the various combinations of properties in real biological data. We report all directed connectivity estimators were able to identify the correct directional influence in the condition where theta oscillation in the HPC is replaced by the ongoing SuM theta oscillations (BP condition). The correct identification of imposed causality is reproducible across two different behavioral contexts and can be clearly distinguished from no manipulation. We show that MVAR-based methods are more correlated to coupling strength, but are more susceptible to the influence of SNR and stationarity, whereas PTE and PSI are mostly resistant.

### Methodological considerations

Our main goal was to make use of a unique dataset where directed connectivity is imposed in a biological system with biological “noise,” in order to assess the performance of directed connectivity measures, and how inherent biological signal properties may bias these estimates. In other words, we reversed the conventional process of assessing the properties of DCEs. An obvious consequence to this is that we have no real, deterministic “ground-truth” as a starting point; the directional relationship in our data (BP condition) is assumed, given we externally eliminated HPC theta oscillations and imposed a new one in a quasi-closed loop reinstatement of HPC theta oscillations triggered by SuM theta oscillations. The effectiveness of our manipulations to produce a causal, uni-directional drive from the SuM to HPC is evidenced by similarities between spectral and non-directed connectivity estimates in the BP condition compared to CON.

As mentioned above, we assume that our BP condition provides deterministic drive to the HPC from closed-looped triggering off SuM LFP. Therefore, we assume the non-maximal directed connectivity values are affected by “noise” or, in essence, biological processes that contribute to the stochastic nature of LFPs. Therefore, we did not assess the effects of SNR and stationarity on DCEs in a traditional sense where these qualities are simulated and tested independently in discrete steps to demonstrate their relative impact. Instead, our assessment involves establishing effects of varying coupling strength, SNR and stationarity in a mixed, continuous scale, bounded by biologically defined limits. Our correlation analyses involving coupling strength, SNR and stationarity are therefore not a direct test of how much these quantities independently bias DCEs. In other words, we assume any signs of interdependence between directed connectivity measures and coupling strength, SNR or stationarity to indicate a sensitivity of the former to the latter. The sensitivities to coupling strength, SNR and stationarity may be inter-dependent, as well as depending on hidden variables not examined in the current study.

It should also be noted that the relevance of our findings is limited by the nature of our data. For instance, we used bipolar, intra-cranial electrodes in our recordings. This setup allows us to practically ignore the contribution from volume conduction and contamination from a common source, and consequently assume the effects of linear mixed noise and correlated noise are minimal. The main focus on our data was the BP condition, where data were acquired from swimming rats. In this behavioral state, the HPC and SuM are known to normally exhibit high-amplitude, continuous theta oscillations (see spectrograms in Figure 1). Therefore, our BP condition is essentially comprised of, qualitatively speaking, highly regular and stationary sinusoidal signals. This caveat can be appreciated in our comparisons between BP/FI and BP/WM data collected from the same rats (Figure 6), where BP/FI presumably included a richer behavioral repertoire than swimming alone (in BP/WM), hence more variability in the occurrence, as well as power of recorded theta oscillations. Nevertheless, the goal of this study is to advance the understanding of how real biological signals, instead of simulated data, behave when examined by various signal processing algorithms. We highlight here the importance of considering experimental and recording conditions when choosing which analyses to apply.

Finally, our paired HPC-SuM recordings yielded a conceptually and computationally less complex bivariate system (as opposed to a multivariate system) to explore their interactions. Given the lack of true multivariate implementation of PTE and PSI, our bivariate data was particularly suitable for a direct comparison between directed connectivity estimators examined here. However, we note that human EEG/fMRI datasets are rarely bivariate, and studies in non-human animals are increasingly more complex with multi-site recordings. Generally, a multivariate approach with multivariate data is superior, under the assumption that all signal sources of interest are functionally connected; an approach that can explain more variance in the system can lead to better insight and interpretation of complex data. Given our experimental setup, our work is only directly applicable to a bivariate system. Direct generalization to multivariate systems is unwarranted and needs further exploration.

### Relationship between data, directed connectivity estimators, and their performances

The key finding from our analyses is that all the DCEs correctly identified the imposed directionality in the BP condition. However, it is also clear that the estimations for all other conditions were not consistent, and some defy our current understanding of the circuit. The relevance of current findings to the underlying neural circuitry will be discussed in a later section. In this section, we will briefly discuss the relationship between our data and directed connectivity estimates.

In general, directed connectivity estimators in the same “family” expectedly yielded similar results in the frequency and time domains. Particularly, the MVAR-based methods are largely in high correspondence, with the exception of DTF in CON and REG conditions. It is not immediately clear why DTF produced relatively substantial different estimate different scores in the frequency domain. In the CON condition, the dominant frequency of DTF estimates is shifted to a higher frequency compared to all other estimates (see Figure 3A), which may be the source of the lack of frequency-domain correspondence. In the REG condition, the imposed 7.7 Hz stimuli essentially act as a potential common source, or a source that indirectly drives SuM activity via the HPC (Swanson and Cowan, 1979). Given the proposition that DTF has better frequency resolution (Blinowska, 2008), it is possible these properties of DTF resulted in markedly different frequency representations in CON and REG conditions. Visual inspection of the frequency spectra from DTF outputs supports this interpretation, where sharper peaks can be observed within the theta band compared to all other methods (data not shown). Nonetheless, there is high correspondence of averaged theta directed connectivity across all MVAR-based methods in the time domain, suggesting that averaged theta DTF estimates was still largely consistent with all other MVAR-based estimates.

It has been shown that in the presence of (simultaneous) bi-directional interaction, PSI is not able to accurately assess the direction of causal influence (Vinck et al., 2015). We have not extensively tested the existence and magnitude of bi-directionality in our bivariate HPC-SuM system in our various experimental conditions. However, we do note that the only condition in our experimental settings that is unidirectional would be the BP condition, where the signal of interest (theta oscillations) is externally imposed. Conservatively, we can conclude that PSI correctly identifies the unidirectional causal influence from the SuM to HPC as per our manipulations. It is unclear if PSI in other conditions offers an accurate estimate due to its dependency on interactions being uni-directional.

PTE is the only non-linear approach examined here. Given the conceptual similarity between TE and Granger causality in general, it has been proposed both approaches essentially estimate the same underlying property under some conditions (Barnett et al., 2009; Seghouane and Amari, 2012). We expected GGC being Granger causality in the frequency domain would yield similar estimates compared to PTE, which was implemented essentially as TE in the frequency domain. However, this was not the case as PTE shared more similarities with PDC and DTF in the frequency domain compared to GGC, and didn't show more correspondence to GGC in the time domain. There are only a few studies employing PTE (Lobier et al., 2014; Dauwan et al., 2016; Hillebrand et al., 2016; Engels et al., 2017), and to the best of our knowledge, no direct performance comparisons have been made between PTE and other directional measures. The original that introduced the technique concluded that PTE can detect bidirectional interactions. However, it was based on simulated data where the interaction occurred at different frequencies for different directions (Lobier et al., 2014). It is not clear how PTE may perform when there are bidirectional interactions at the same frequency range in a bivariate system, and if the estimates may be biased by indirect influences from additional sources unaccounted for—both properties are present in our data based on our current understanding of the HPC-SuM circuit.

### Coupling strength, noise and stationarity

As expected, coupling strength as measured by coherence and PPC were variables that showed high correlation with directed connectivity estimates. Particularly, coherence showed marginally higher correlations compared to PPC. This was expected as the MVAR estimators utilize coherence as part of their calculations, and that both amplitude and phase coupling are taken into account. In contrast, PTE and PSI are nominally amplitude-independent, given only phase information is considered in PTE and only the imaginary part of the coherence is used in PSI. Interestingly, although PTE is considered to be a non-linear measure and uses only phase information for its calculation, we found the magnitude of PTE is correlated with ordinary coherence more than PPC. Conceptually, higher PPC indicates less information content as HPC and SuM phase differences are “stationary” and presumably less variable. It has been noted previously that the amplitude of the signal does in fact affect the estimation of linear phase-coupling (Muthukumaraswamy and Singh, 2011). These factors may have contributed to how the magnitude of PTE is preferentially correlated to coherence over PPC. It is sufficient for the purpose of this study to show that PTE estimates from the direction of causal flow (i.e., SuM to HPC) positively correlate with coupling strength, and the causal flow from the opposite direction (i.e., HPC to SuM) can be negatively correlated to coupling strength as reported previously (i.e., Figure 7A in Lobier et al., 2014). Finally, we show that although PSI is correlated to coupling strength, these correlations were much less than *r* = 0.1. There is no directly comparable simulation in the literature examining the effect of coupling strength with PSI. Based on our data and the lack of mathematical dependencies between PSI, coherence and PPC calculations, we conclude PSI is weakly, if at all, correlated to coupling strength.

None of our measures of SNR and stationarity were found to considerably impact the magnitude of imposed directionality (all Pearson's *r* assessed were below 0.1). However, we did find weak dependencies that were consistent across all estimators, except for PSI, within theoretical expectations. For SNR, the expectation would be that higher SNR lead to better estimation, and indeed most of the estimators, except for PSI, yielded higher magnitude of SuM to HPC direction of theta modulation with higher overall SNR. It is also known that differences in SNR levels across assessed signals may result in incorrect inference of true causality (Bastos and Schoffelen, 2015), given that the signal with lower SNR would have poorer predictive capabilities. Most of the estimators also showed dependencies on the difference ratio of SNRs, suggesting unequal SNR (or, the presence of a weaker node) affects the magnitude of directed connectivity estimation, favoring the signal with higher SNR. The relative immunity of PSI to examined signal properties was expected as the means for directionality detection via PSI does not depend on the ability for each signal to contribute to the other's prediction through various decompositions in time, frequency or phase, but is pragmatically dependent on phase lags and their signs (Nolte et al., 2008).

Stationarity is not a property that has been actively explored in its contributing role to bias directed connectivity estimates; certainly, a central assumption in (biological) signal processing is that the signal is stationary for a given algorithm to return meaningful results. For MVAR-based methods, the common practice is to detrend the input data to make the input *mean* stationary before model order and parameters are computed. As for *variance* stationarity, it is not entirely clear how they may affect directed connectivity estimates. It is unclear in our data, or biological data in general, if the effects of heteroscedasticity can be independent from SNR. Given that heteroscedasticity essentially represent the waxing and waning of LFPs in our context, if we assume that the background “noise” is constant, the time-dependent variations in LFP essentially mirror time-dependent variations in SNR. In this scheme heteroscedasticity essentially interacts with background noise to affect the phase estimation in a non-linear fashion in real data (e.g., Nolte et al., 2004). This may be why PTE, which should be insensitive to heteroscedasticity, display a weak relationship with it as it does with SNR measures.

How heteroscedasticity actually influences directed connectivity is beyond the scope of the current study. In theory, the MVAR process should account for most of heteroscedasticity through the error term. However, variance non-stationarity may affect the accuracy of the model order estimation, particularly in the application of windowed analyses. Our use of step-wise auto-regressive modeling and a constant model order for a given behavioral epoch may have contributed to sub-optimal model order across data segments. As for PTE, the input data are band-passed, Hilbert-transformed time-series. We assume heteroscedasticity affects the SNR, hence the accuracy of phase estimation through Hilbert transform to contribute to PTE sensitivity to variance non-stationarity. Systematic investigation is needed to fully explore the range where heteroscedasticity may significantly bias practical directed connectivity estimates.

We also examined the relationship between directed connectivity estimates and periodicity. We find little evidence that periodicity of the signal affects the determination of directed connectivity, given the low correlation between the two. There is also no difference between how well periodicity correlates with directed connectivity estimates in either direction, suggesting no bias toward the dominant coupling direction. However, as mentioned, our data of main interest were collected from HPC and SuM LFP recorded in the water maze, where persistent high-amplitude theta oscillations accompany vigorous swimming. The dynamic range of periodicity of our data, as measured by the decay constant associated with the auto-correlation function, may not be sufficient to provide an adequate test for the association between periodicity and directed connectivity.

### Implications for SuM-MS-HPC physiology

With non-directed connectivity analyses, we found equivalent HPC and SuM theta power and coherence/PPC in CON and BP conditions, whereas coherence/PPC remained low for other conditions. Previous work has suggested a stationary, 7.7 Hz stimulus train can partially rescue water maze learning (McNaughton et al., 2006). The present data suggest that this partial rescue is likely to be independent of HPCxSuM theta coherence, given coherence/PPC in the REG condition is statistically indistinguishable from TET and IRR and much lower than CON and BP. Additionally, SuM theta PSD was also found to be uncoupled to water maze learning (i.e., lowest in REG and equivalent to CON in TET and IRR), suggesting water maze learning may also be relatively independent of SuM theta oscillations, despite reported experience-dependent changes (Ruan et al., 2011; Hernandez-Perez et al., 2015). While SuM inactivation moderately impairs water maze learning in single-day (Pan and McNaughton, 1997) or multi-day (Shahidi et al., 2004) versions of the task, lesion of SuM does not appear to affect water maze learning in the multi-day version of the task (Pan and McNaughton, 2002; Aranda et al., 2006). Interestingly, serotonergic depletion in the SuM also leads to water maze learning deficits in the multi-day version of the task (Hernandez-Perez et al., 2015). Taken together, it appears SuM involvement in water maze learning is minor, and is relatively independent of local theta activities.

Our hypotheses regarding how the SuM-MS-HPC circuit would respond to our perturbations were not fully supported. The unanimous agreement of SuM to HPC direction of theta modulation in the BP condition was the most crucial finding in our attempt to test the DCEs with real biological data, and is consistent with our expectations from given manipulations. The second most consistently identified direction of causal influence is SuM to HPC under the TET condition. While it is possible that MVAR-based methods were affected significantly by unequal SNR, favoring a SuM to HPC direction of theta modulation due to the lack of SNR in the HPC (Bastos and Schoffelen, 2015; Vinck et al., 2015), it is also biologically plausible to assume that since MS-inactivation did not completely eliminate HPC theta or HPC-SuM theta coherence, the remaining HPC theta oscillations may in fact be driven by the SuM.

The direction of theta modulation was not expected to be different between TET and IRR conditions, given the latter was not designed to evoke activities at theta frequencies. The hypothesis was mostly supported, as all but PSI indicated a SuM to HPC direction of theta modulation. In addition, the magnitude of the SuM to HPC drive was marginally lower than those observed for TET (except for PTE). This is consistent with the observation that there was lower HPC theta power and lower HPC-SuM theta coherence in the IRR condition, either due to group differences or disruption of ongoing HPC theta oscillations through irregular stimulation of the fornix.

Given direct experimental evidence and previous reports on Granger causality interactions between the SuM and HPC (Kocsis and Kaminski, 2006; Ruan et al., 2011), it was expected that regular stimulation of the fornix at 7.7 Hz would not only elicit stimulus-bound theta activities in the HPC, but also modulate SuM theta LFPs. Instead, we observed the lowest theta power in the SuM in the REG condition, while the HPC oscillated at driven 7.7 Hz at power comparable to CON and BP conditions. Theta coherence was also the lowest for REG compared to all other conditions. Again, we cannot rule out group differences contributing to some of the differences between directed connectivity estimates. Regardless, our data do suggest that HPC to SuM modulation may not be theta-rhythmic as we and others originally assumed (Kocsis and Kaminski, 2006; Ruan et al., 2011); otherwise spectral power at 7.7 Hz should have been observed in the SuM, and HPCxSuM theta coherence at that frequency should also have been detected. Alternatively, our administration of tetracaine may have affected the neighboring lateral septum, blocking the presumed feedback pathway from HPC to SuM (Swanson and Cowan, 1979). However, the presence of a theta harmonic in the SuM, and its elevated coherence with the HPC in the BP condition does indicate rhythmic feedback from the HPC may reach the SuM in our experimental setting. It is not possible to conclusively determine if putative HPC feedback to the SuM is theta rhythmic or not with our current data. Nevertheless, directed connectivity estimates were the most divergent, with PTE returning statistically significant estimation of HPC to SuM direction of theta modulation, but statistically significant modulation in the opposite direction was observed for DOCH, GGC and PSI estimates. Since there was no detectable 7.7 Hz power in the SuM PSD, and that SuM theta PSD was low, we assume that the DCOH, GGC and PSI estimations are inaccurate. It is more parsimonious to conclude that there are no HPC-SuM interactions at theta frequencies in the REG condition, given: (1) unlikelihood of a HPC to SuM direction of modulation in the absence of strong SuM theta power and HPCxSuM theta coherence and; (2) the lack of consistency across different DCEs. However, it should be re-iterated that the HPC imposes a downstream frequency-limiting effect on SuM theta rhythmic bursting activity in anaesthetized rats (Kirk et al., 1996; Kirk, 1997; Kocsis and Kaminski, 2006) and LFP in freely moving rats (Ruan et al., 2017). An imposed, relatively higher frequency (7.7 Hz) HPC theta may non-rhythmically suppress SuM theta PSD. It is unclear if an increase of slower theta (<5 Hz)/delta activities seen in SuM spectrum in the REG condition is also a consequence of our 7.7 Hz stimulation.

In the CON condition, where no manipulations exist, PDC, DTF, DCOH, PTE, and PSI did not detect a bias in the direction of theta modulation. Only GGC favored the SuM to HPC direction and PSI supported modulation in the opposite direction. Our previous data suggested that there may be a gradual modification of the direction of HPC and SuM theta coupling during the course of WM learning, demonstrating HPC and SuM interaction is dynamic and can be modified by experience (Ruan et al., 2011). The most parsimonious interpretation of conflicting directed connectivity difference scores appears to be that neither HPC nor SuM appear to preferentially drive the other. Given the non-directed connectivity (coherence and PPC) is high in the CON condition, we interpret the lack of preferential driving to be balanced bi-directional interactions, rather than a total lack of functional coupling between HPC and SuM at theta frequencies as in REG.

## Concluding remarks

Here, we used real biological data with experimentally driven causality to assess the performance of various directed connectivity estimators. Our unique approach indicates that all DCEs are able to clearly identify imposed uni-directional interactions across different behavioral contexts in a bivariate system. Further, our correlation analyses corroborate with simulation studies where levels of coupling strength, SNR and variance stationarity all make modest potential contributions to bias estimates from MVAR-based methods, whereas non-MVAR methods are relatively unaffected. Comparisons made in the current study provide the crucial missing link between theory and practice, where the use of simulated data do not fully recapitulate the complexity of real biological data. Our analyses also support recommendations made elsewhere, that methods developed to tackle the problem of directed connectivity all have weakness and strengths; using a selection of different methods should be considered if the nature of the system and the inherent properties of input signals are not unequivocally understood (Wang et al., 2014; Bastos and Schoffelen, 2015).

## Author contributions

CY conceived the study, carried out part of the histology and analyzed the data. MR collected the behavioral, neurophysiological and histological data. CY and NM prepared the manuscript.

### Conflict of interest statement

NM has a confidential disclosure and consulting agreement with Janssen Research & Development, LLC that does not constitute a conflict. The other authors declare that the research was conducted in the absence of any commercial or financial relationships that could be construed as a potential conflict of interest.
